# After Nerve Injury, Lineage Tracing Shows That Myelin and Remak Schwann Cells Elongate Extensively and Branch to Form Repair Schwann Cells, Which Shorten Radically on Remyelination

**DOI:** 10.1523/JNEUROSCI.1453-17.2017

**Published:** 2017-09-13

**Authors:** Jose A. Gomez-Sanchez, Kjara S. Pilch, Milou van der Lans, Shaline V. Fazal, Cristina Benito, Laura J. Wagstaff, Rhona Mirsky, Kristjan R. Jessen

**Affiliations:** Department of Cell and Developmental Biology, University College London, Gower Street, London WC1E 6BT, United Kingdom

**Keywords:** injury, nerve, PNS, remyelination, regeneration, Schwann cell

## Abstract

There is consensus that, distal to peripheral nerve injury, myelin and Remak cells reorganize to form cellular columns, Bungner's bands, which are indispensable for regeneration. However, knowledge of the structure of these regeneration tracks has not advanced for decades and the structure of the cells that form them, denervated or repair Schwann cells, remains obscure. Furthermore, the origin of these cells from myelin and Remak cells and their ability to give rise to myelin cells after regeneration has not been demonstrated directly, although these conversions are believed to be central to nerve repair. Using genetic lineage-tracing and scanning-block face electron microscopy, we show that injury of sciatic nerves from mice of either sex triggers extensive and unexpected Schwann cell elongation and branching to form long, parallel processes. Repair cells are 2- to 3-fold longer than myelin and Remak cells and 7- to 10-fold longer than immature Schwann cells. Remarkably, when repair cells transit back to myelinating cells, they shorten ∼7-fold to generate the typically short internodes of regenerated nerves. The present experiments define novel morphological transitions in injured nerves and show that repair Schwann cells have a cell-type-specific structure that differentiates them from other cells in the Schwann cell lineage. They also provide the first direct evidence using genetic lineage tracing for two basic assumptions in Schwann cell biology: that myelin and Remak cells generate the elongated cells that build Bungner bands in injured nerves and that such cells can transform to myelin cells after regeneration.

**SIGNIFICANCE STATEMENT** After injury to peripheral nerves, the myelin and Remak Schwann cells distal to the injury site reorganize and modify their properties to form cells that support the survival of injured neurons, promote axon growth, remove myelin-associated growth inhibitors, and guide regenerating axons to their targets. We show that the generation of these repair-supportive Schwann cells involves an extensive cellular elongation and branching, often to form long, parallel processes. This generates a distinctive repair cell morphology that is favorable for the formation of the regeneration tracks that are essential for nerve repair. Remyelination, conversely, involves a striking cell shortening to form the typical short myelin cells of regenerated nerves. We also provide evidence for direct lineage relationships between: (1) repair cells and myelin and Remak cells of uninjured nerves and (2) remyelinating cells in regenerated nerves.

## Introduction

Regeneration of injured nerves depends on the Schwann cell injury response, namely a switch in Schwann cell phenotype from the myelin and Remak (non-myelin-forming) states before injury to a cell specialized to support regeneration, the repair Schwann cell (for review, see Jessen et al., 2015a,2015b, 2016). This cell, which occupies the nerve distal to the injury site, supports the survival of axotomized neurons, promotes axonal regeneration, organizes myelin clearance, and forms regeneration tracks (bands of Bungner) that guide axons back to their targets (for review, see Zochodne, 2012; Glenn and Talbot, 2013; Scheib and Höke, 2013; Brosius Lutz and Barres, 2014).

Defining the biology of repair cells is central to understanding the mechanisms of nerve regeneration and how repair can be facilitated. It is therefore surprising that one of the basic parameters of repair cells has remained obscure: their structure. Therefore, the structural transformations when myelin and Remak cells form repair cells and when repair cells myelinate after regeneration have remained obscure. Although these morphological switches are a central feature of nerve repair, little effort has been spent investigating the mechanisms that control them, such as changes in the cytoskeleton and adhesion molecules. Furthermore, although there is consensus that these conversions take place, namely that Remak and myelin cells must be the direct source of the repair cells seen in Bungner's bands of injured nerves and that these cells can convert to myelin cells after regeneration, there has been no direct demonstration of these lineage relationships using lineage-tracing methods

Historically, the morphology of repair cells has been characterized as elongated, bipolar, and inferred from their appearance in transverse sections of the Bungner bands that characterize nerves distal to injury (Weinberg and Spencer, 1978). These are columns made from overlapping repair cells, lying inside the basal lamina tubes that previously enclosed myelin or Remak cells and their associated axons before injury. Repair cells organized into Bungner bands are essential for axonal guidance during regeneration, unlike development, during which axons can reach their target fields in the absence of glial cells (Schwann cell precursors and immature Schwann cells) (Grim et al., 1992; Riethmacher et al., 1997).

The generation and maintenance of repair cells involves transcriptional mechanisms that are not important for developing Schwann cells, including c-Jun, STAT3, andH3K27 trimethylation (Parkinson et al., 2008; Arthur-Farraj et al., 2012; Jessen and Mirsky, 2016; Ma and Svaren, 2016; Benito et al., 2017). Signaling mechanisms activated after injury, such as the P38, JNK, and Erk1/2 MAP kinase pathways and gpr126 and histone deacetylase 2 (HDAC2), have also been studied (for review, see Boerboom et al., 2017). Schwann-cell-derived trophic factors, which potentially support regeneration have been examined (for review, see Boyd and Gordon, 2003; Chen et al., 2007; Scheib and Höke, 2013; Wood and Mackinnon, 2015) and mechanisms of myelin clearance including myelinophagy and macrophage recruitment have been identified (Hirata and Kawabuchi, 2002; Gomez-Sanchez et al., 2015; Suzuki et al., 2015).

Here, we have used mouse models engineered for genetic lineage-tracing and block-face-scanning electron microscopy to provide the first description of the length and structure of repair cells at different times after nerve transection and to compare them with immature, myelin, and Remak Schwann cells. Unexpectedly, this revealed that the conversion of myelin and Remak cells to repair cells involves a 2- to 3-fold increase in cell length, generating cells that are 7- to 10-fold longer than immature Schwann cells in developing nerves. The elongation of repair cells is often accompanied by branching to form long, parallel processes. This lengthening is strikingly reversible because repair cells shorten to ∼1/7 of their length as they remyelinate regenerated axons. We also provide the first direct lineage-tracing evidence for two important tenets in Schwann cell biology: (1) the conversion of myelin and Remak cells in uninjured nerves to the denervated Schwann cells in Bungner's bands and (2) the conversion of these repair cells back to myelin cells after nerve regeneration.

## Materials and Methods

### 

#### 

##### Transgenic mice.

Animal experiments conformed to UK Home Office guidelines and were supervised by UCL Biological Services. To obtain inducible repair Schwann cells, we crossed proteolipid protein (PLP)-CreERT2^+/−^ (MGI: 2663093) or P0Cx-CreERT2^+/−^ (MGI: 2663097) mice (Leone et al., 2003) with Rosa26stop-EYFP^fl/fl^ mice (The Jackson Laboratory, catalog #006148, MGI: 2449038) and back-crossed these with Rosa26stop-EYFP^fl/fl^ mice to obtain PLPCreERT2^+/−^EYFP^fl/fl^ or P0Cx-CreERT2^+/−^EYFP^fl/fl^ offspring.

Tamoxifen (Sigma-Aldrich) was dissolved in sunflower oil (Sigma-Aldrich) and absolute ethanol (10:1). The mice received a single injection of 5–10 mg/kg tamoxifen at 28 (P0Cx-CreERT2) or 42 (PLP-CreERT2) days after birth. To produce embryo samples, pregnant females were injected with 5 mg/kg at embryonic day 12.5 (E12.5) to obtain labeled immature Schwann cells at E18. Tamoxifen injection activates the ERT2 receptor, causing the active Cre-recombinase to remove the stop codon between *loxP* sites that results in the expression of *YFP* located at the *Rosa26* locus.

##### Mouse genotyping.

DNA for genotyping was extracted from ear or tail samples applying the Hot Shot method (Truett et al., 2000). Primers used to genotype for the *PLP-CreERT2* transgene were 5-CAC TCT GTG CTT GGT AAC ATG G-3′ and 5′-TCG GAT CCG CCG CAT AAC C-3′ (520bp); for the *P0Cx-CreERT2* transgene were 5′-CTG CAC AGA CAT GAG ACC ATA GG-3′ and 5′-TCG GAT CCG CCG CAT AAC C-3′ (800bp); for the *Cre* sequence were 5′^-^ACC AGG TTC GTT CAC TCA TGG-3′ and 5′-AGG CTA AGT GCC TTC TCT ACA-3′ (200bp); and for the *YFP* transgene were 5′-AAG ACC GCG AAG AGT TTG TC-3′, 5′-AAA GTC GCT CTG AGT TGT TAT-3′ and 5′-GGA GCG GGA GAA ATG GAT ATG-3′ (320 bp in the homozygous mouse and two bands at 320 and at 600 bp in the heterozygous mouse).

##### Nerve injury.

Mice were anesthetized with isoflurane and, at 60 d after birth, the sciatic nerves of adult mice were cut at the sciatic notch. To avoid axonal growth inside the distal stump and regeneration, the proximal stump was tied back to the triceps muscle. To analyze the repair Schwann cells at different time points after injury (1, 4, and 10 weeks and 6 months) mice were killed and the injured tibial nerve and the intact nerves of the contralateral side were obtained for whole-mount labeling, immunohistochemistry, or electron microscopy analysis.

##### Immunolabeling of teased nerves.

Nerves were dissected and fixed in 4% paraformaldehyde/PBS for 4 h at 4°C. The fibers were gently teased apart using a dissecting microscope and left to dry for 60 min at room temperature, blocked in 5% BSA, 0.2% Triton X-100 in TBS for 30 min, and incubated in primary antibodies overnight at room temperature. The primary antibodies were anti-green-fluorescent (anti-GFP) protein (1:500 chicken polyclonal; Invitrogen Life Technologies, catalog #A10262) and anti-c-Jun (1:200 rabbit polyclonal; Cell Signaling Technology, catalog #9165). After incubation, fibers were rinsed with PBS and then the samples were reprobed with Alexa Fluor 488 anti-chicken antibodies (1:500, Invitrogen Life Technologies, catalog #A11039), donkey anti-rabbit Cy3 antibodies (1:500, Jackson ImmunoResearch, catalog #711-165-152), and DAPI diluted 1:20.000 in blocking buffer for 2 h at room temperature. In other experiments, labeling with anti-GFP antibodies and Alexa Fluor 488 anti-chicken antibodies was followed by 1 h incubation with anti-L1 (1:10 rat 324 anti-mouse Ig 1:10, Merck, catalog #MAB5272) and 1 h incubation with Cy3 anti-rat (1:500, Jackson ImmunoResearch, catalog #112-165-003). The nerves were mounted in Fluoromount G (Southern Biotech).

##### Immunolabeling of whole-mount preparations.

Nerves, including embryo nerves, were dissected and fixed in 4% paraformaldehyde/PBS for 5 h at 4°C. Then nerves were permeabilized 3 times for 10 min each at room temperature in PTX (PBS 1× containing 1% Triton X-100), followed by incubation overnight with blocking buffer (PBS containing 10% FBS). Afterward, nerves were probed overnight at 4°C with rabbit anti-GFP primary antibody at a concentration of 1:1500 diluted in blocking buffer (rabbit polyclonal; Invitrogen Life Technologies, catalog #A6455). After incubation, nerves were rinsed with PTX 3 times for 5 min each, followed by washing in PTX for 6 h at 4°C on the shaker, changing the PTX every hour. Then samples were reprobed with donkey anti-rabbit Cy3 antibody diluted 1:1000 (Jackson ImmunoResearch, catalog #711-165-152) and DAPI diluted 1:20.000 in blocking buffer for 48 h at 4°C on the shaker. The tissue was then washed in PTX 3 times for 15 min each, then for 6 h, changing the PTX each hour, and subsequently in PTX overnight. To facilitate the embedding of nerves in CitiFluor, nerves were cleared stepwise in 25%, 50%, and 75% glycerol in PBS. Finally, nerves were mounted in CitiFluor mounting medium and aligned longitudinally.

##### Confocal microscopy.

Whole-mount images were recorded with a 25×/0.95 Leica objective water-immersion lens (∞/0.17/D HCX IRAPO), with 0.6 μm step size and a resolution of 1024 × 1024 pixels using a Leica TCS SP8 confocal microscope. Schwann cell length and morphologies were measured by the freehand measurement tool of IMARIS version 8.4 software (Bitplane), allowing measurement of all types of Schwann cells (myelin, Remak, and repair Schwann cells) in the tibial nerve. Only Schwann cells in which the beginning and end of the structure could be clearly determined were chosen. For each time point after injury, a variable number of Schwann cells was measured from 6–10 mice at each time point (Table 1).

**Table 1.** Cell length

	Length (μm)	SD (μm)	SEM (μm)	Cells (*n*)	Mice (*n*)
PLP-CreERT2					
Immature Schwann	112.6	33.9	3.60	89	9
Remak Schwann	256.1	96.5	4.60	440	10
Repair Schwann 7 d after cut	522.1	225.6	20.51	121	8
Repair Schwann 4 wk after cut	760.9	275.5	23.12	142	7
Repair Schwann 10 wk after cut	519.2	226.4	19.41	136	10
Repair Schwann 6 mo after cut	379.1	150.8	28.01	29	5
P0Cx- CreERT2					
Myelin Schwann	575.9	116.2	11.51	102	7
Repair Schwann 4 wk after cut	1117.0	384.8	43.02	80	9
Remyelinating Schwann 6 wk after grafting	141.1	42.2	6.21	62	10

##### Electron microscopy.

Nerves taken at 4 weeks after injury were dissected and fixed in 2% paraformaldehyde and 2.5% glutaraldehyde in 0.15 m cacodylate buffer, pH 7.4, with 2 mm calcium chloride overnight at 4°C. The nerves were then processed following the National Center for Microscopy and Imaging Research methods for 3D electron microscopy, a protocol for preparation of biological specimens for serial block-face-scanning electron microscopy (www.ncmir.ucsd.edu/sbem-protocol/). Tissues were washed 5 times for 3 min in cold 0.15 m cacodylate buffer containing 2 mm calcium chloride, followed by a 1 h incubation in 2% OsO_4_ and 3% potassium ferrocyanide in H_2_O buffer. After rinsing in ddH_2_O, samples were placed in thiocarbohydrazide solution for 20 min, followed by a second exposure to 2% OsO_4._ They were then placed in 1% uranyl acetate in H_2_O at 4°C overnight and then incubated in lead aspartate in H_2_O solution at 60°C for 30 min, after which tissues were dehydrated in a serial washes of ethanol and acetone. Nerves were embedded in Durcupan ACM resin (Fluka).

Stacks of backscatter electron micrographs were automatically acquired using a Gatan 3View system in conjunction with a Zeiss field emission scanning electron microscope. At a standard resolution of 1000×, the total area sampled measured 255.4 μm^2^ on *x* and *y* and between 100 and 150 μm on *z*. The resulting stacks were normalized for contrast and brightness and converted to TIFF images in Digital Micrograph before importation in Amira 5.3 software (FEI; Thermo Fisher Scientific) for tracing and manipulation. Individual Bungner bands, single cells within them, and myelin debris within individual Schwann cells were traced using the drawing tool and transformed into 3D projections for presentation.

##### Statistical analysis.

Data are presented as the arithmetic mean ± SEM. Statistical significance was estimated by one-way ANOVA with Tukey correction. *p* < 0.05 was considered statistically significant. Statistical analysis was performed using GraphPad Prism software version 6.0.

## Results

**Figure 1.**
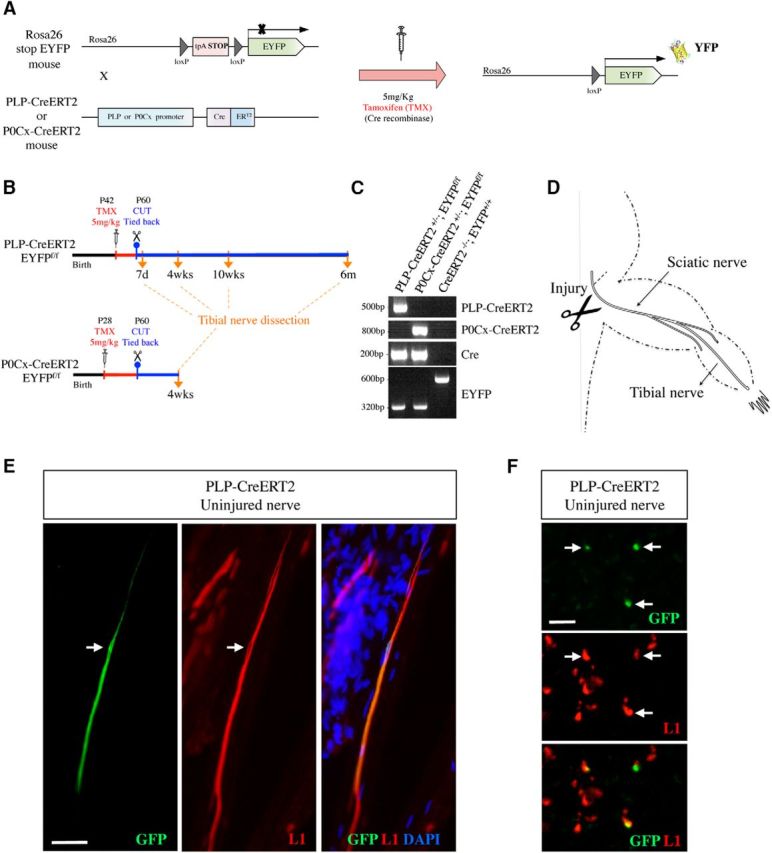
Mouse generation, experimental outline, and cell-type specificity of genetic labeling. ***A***, Generation of PLP-Cre ERT2xRosa26stopEYFP and P0Cx-Cre ERT2xRosa26stopEYFP mice. ***B***, Outline of lineage-tracing experiments involving tamoxifen injection of uninjured mice followed by nerve transection without reinnervation. ***C***, PCR analysis showing the presence of the expected PCR products in the mouse lines indicated. ***D***, Diagram showing the experimental nerves and the site of nerve transection. ***E***, ***F***, In uninjured nerves of tamoxifen-treated PLP-Cre ERT2xRosa26stopEYFP mice, GFP expression is essentially restricted to Remak cells. ***E***, Double immunolabeling of a teased nerve with GFP and L1 antibodies. Middle, Schwann cells in a Remak bundle identified by L1 expression. One of the cells expresses GFP (left). Arrow indicates the nucleus. ***F***, Double immunolabeling of transverse cryostat sections using GFP and L1 antibodies. The three GFP-positive cells (top) also express L1 (middle). Scale bars: ***E***, 50 μm; ***F***, 50 μm.

To visualize Schwann cells in developing, adult, and injured nerves we used two mouse lines, PLP-CreERT2xRosa26stopEYFP and P0Cx-CreERT2xRosa26stopEYFP, in which yellow fluorescent protein (YFP) is expressed in Schwann cells after tamoxifen administration (Fig. 1*A*,*C*). Relatively low doses of tamoxifen were used because we aimed to activate YFP expression in relatively few cells for clear visualization of individual cells. The selective expression of YFP in myelin Schwann cells of adult uninjured nerves in tamoxifen-treated P0Cx-CreERT2xRosa26stopEYFP mice has been reported previously (Ribeiro et al., 2013), a finding that we confirmed (data not shown). The expression of YFP in Remak cells in uninjured nerves of tamoxifen-treated PLP-CreERT2xRosa26stopEYFP mice was verified using both teased nerves and frozen transverse nerve sections. Double labeling of these preparations with L1 antibodies to identify Remak cells (Martini, 1994) and GFP/YFP antibodies showed that GFP/YFP labeling was essentially restricted to L1-positive cells (Fig. 1*E*,*F*), although a few cells identified as myelin cells by morphology also expressed GFP (see below).

### Immature Schwann cells in uninjured developing nerves

**Figure 2.**
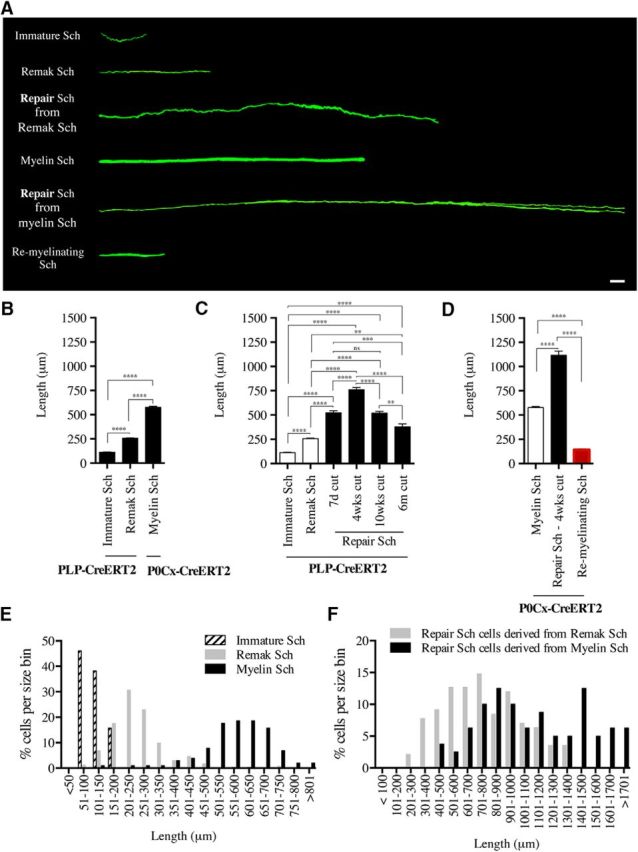
Synopsis of the appearance and length of Schwann cells in developing, uninjured, injured, and remyelinating nerves. ***A***, Direct comparison of the cell types visualized in the present work, showing representative examples of each cell. All cells are magnified equally. Scale bar, 30 μm. ***B***, Length of immature, Remak, and myelin Schwann cells in developing and adult uninjured nerves. Measurements of immature and Remak cells are from PLP-Cre ERT2xRosa26stopEYFP mice. Measurements of myelin cells are from P0Cx-Cre ERT2xRosa26stopEYFP mice. Immature cells, *n* = 89; Remak cells, *n* = 440; myelin cells, *n* = 102. ****p < 0.0001, one-way ANOVA, Tukey's comparison. ***C***, Length of denervated Schwann cells (repair cells) derived from Remak cells. The graph shows cell length at different times after injury without reinnervation. Measurements are from PLP-Cre ERT2xRosa26stopEYFP mice. The length of immature and Remak cells in uninjured nerves of the same mouse line is shown for ease of comparison (white columns). Note that, at 4 weeks, repair cells are several-fold longer than immature and Remak cells and that long-term denervation is accompanied by a significant reduction in cell length. Seven-day cut, *n* = 121; 4-week cut, *n* = 142; 10-week cut, *n* = 136; 6-month cut, *n* = 29. **p < 0.01; ***p < 0.001; ****p < 0.0001, one-way ANOVA, Tukey's comparison. ***D***, Length of repair cells derived from myelin cells in 4-week cut nerves and the length of myelin cells that have remyelinated regenerated axons and are derived from repair cells in the same mouse line, P0Cx-Cre ERT2xRosa26stopEYFP. The length of myelin cells before injury is shown for ease of comparison (white columns). Repair cells, *n* = 80; remyelinating cells, *n* = 62. ****p < 0.0001, one-way ANOVA, Tukey's comparison. ***E***, Size distribution of immature Schwann cells (Sch), Remak Schwann cells, and myelin Schwann cells in uninjured nerves. ***F***, Size distribution of repair cells derived from Remak Schwann cells and from myelin Schwann cells.

**Figure 3.**
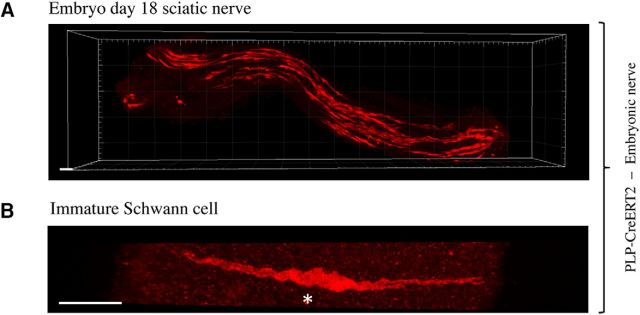
Immature Schwann cells in uninjured nerves. ***A***, Low-magnification view of a nerve segment from an E18 embryo of a PLP-Cre ERT2xRosa26stopEYFP mouse showing numerous immature Schwann cells distributed along the nerve. Scale bar, 30 μm. ***B***, Single E18 Schwann cell showing a typical wavy structure. Asterisk indicates the position of the nucleus. Scale bar, 30 μm.

To examine immature Schwann cells, we used PLP-CreERT2xRosa26stopEYFP mice because PLP is expressed in Schwann cell precursors and immature Schwann cells (Leone et al., 2003; Le et al., 2011). Sciatic nerves were removed from E18 embryos of pregnant mice injected with tamoxifen (5 mg/ml) 5.5 d earlier. After processing for GFP/YFP immunohistochemistry, the nerves were examined by confocal microscopy and the length of individual cells was measured using the freehand tool of IMARIS software. Examination at low magnification showed several elongated cells scattered throughout the nerves (Figs. 2*A*,*B*, 3*B*,*C*). Electron microscopy suggests that, at this stage, most Schwann cells have the structure of irregular flattened sheets that stretch along and around groups of axons that they envelop communally (Webster et al., 1973). Although the details of these features were not revealed in the light microscope, the relatively broad structure of the cells and generally rather diffuse aspect of the fluorescence signal is consistent with this morphology. The cells were unbranched and most of them showed an undulating, wave-like shape, which is likely to reflect their normal appearance because the nerves were initially fixed *in situ* with the limb in an extended position, followed by postfixation. The average length of immature Schwann cells was 112.6 μm, ranging from 57.8 to 210.0 μm (Fig. 2*A*,*B*, Table 1).

### Remak Schwann cells in uninjured adult nerves

**Figure 4.**
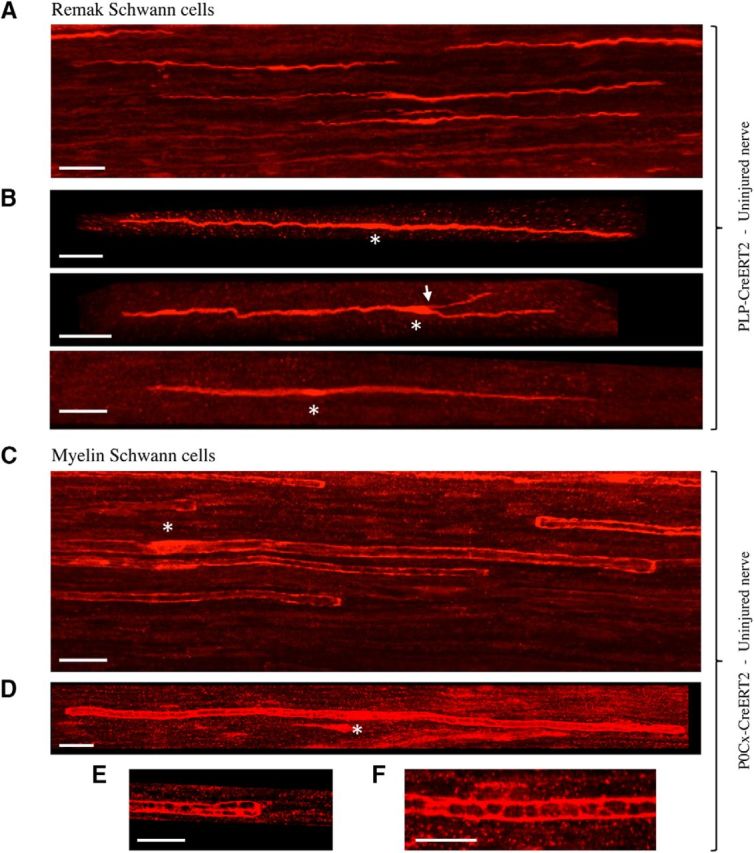
Remak and myelin Schwann cells in uninjured adult nerves. ***A***, Overview showing several scattered Remak cells in the tibial nerve of a PLP-Cre ERT2xRosa26stopEYFP mouse. The cells often have a wavy appearance. Scale bar, 30 μm. ***B***, Higher-magnification view illustrating two typical Remak cells (top and bottom) and a Remak cell with a short branch (arrow, middle). Asterisks indicate position of the nucleus. Scale bar, 30 μm. ***C***, Overview showing a part of a number of myelin cells in the tibial nerve of a P0Cx-Cre ERT2xRosa26stopEYFP mouse. Asterisk indicates position of the nucleus. The cells have a straight appearance and the blunt ends of myelin cells, heminodes, are seen clearly (arrows). As expected, the labeling is strongest in the outer cytoplasmic compartment (longitudinal Cajal bands), generating a railway track appearance. Scale bar, 30 μm. ***D***, Whole myelin cell with heminodes visible at both ends (arrows). Longitudinal Cajal bands are clearly seen, but transverse Cajal bands are indistinct. Asterisk indicates the position of a nucleus. Scale bar, 30 μm. ***E***, Closer view of the end of a myelin cells showing longitudinal and transverse Cajal bands at a heminode. Scale bar, 20 μm. ***F***, Longitudinal and transverse Cajal bands near the middle of a myelin cell. Scale bar, 20 μm.

Tibial nerves of PLP-CreERT2xRosa26stopEYFP mice treated with a low dose of tamoxifen were used to examine adult Remak cells after immunolabeling with GFP/YFP antibodies. We found that 93% of labeled cells were Remak cells and 7% were myelin cells. The preferential expression in Remak cells rather than myelin cells is likely related to the low dose of tamoxifen used in these experiments (Leone et al., 2003). Most commonly, several Remak cells were labeled in each microscope field (Fig. 4*A*). They appeared as narrow, bipolar cells that were generally unbranched and frequently wavy, in contrast to the straight appearance of myelin (see subsequent section; Fig. 4*A*,*B*). Approximately 10% of these cells carried one or two short branches (Fig. 4*B*). Remak cells were just over twice as long as E18 immature Schwann cells, measuring on average 256.1 μm, ranging from 82.0 to 825.0 μm (Fig. 2*A*,*B*, Table 1).

### Myelin Schwann cells in uninjured nerves

Myelin Schwann cells were visualized in the tibial nerves of tamoxifen-treated P0Cx-CreERT2xRosa26stopEYFP mice. Myelin cells showed a characteristic and readily recognizable appearance. They were relatively straight, unbranched, and with larger diameters than Remak cells (Fig. 4*C*,*D*). Heminodes, resulting from one of the two cells forming a node of Ranvier being induced to express YFP, were seen clearly, as were Cajal bands (Fig. 4*C–F*). On average, myelin cells were approximately twice as long as Remak cells, at 575.9 μm, ranging from 138 to 849.0 μm (Fig. 2*A*,*B*, Table 1).

### Repair Schwann cells derived from Remak cells

**Figure 5.**
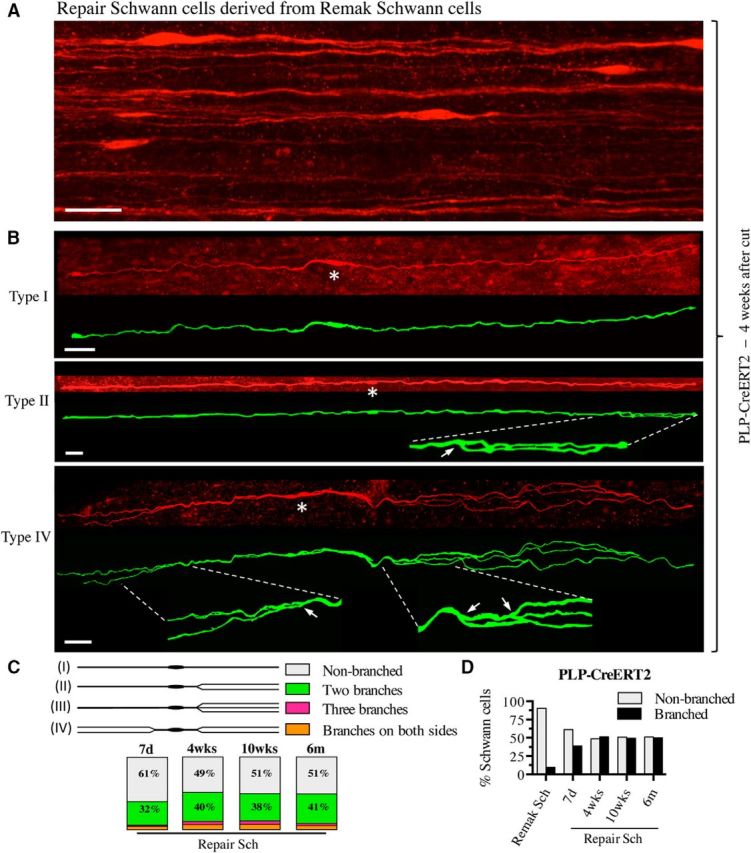
Remak-derived repair cells. ***A***, Overview showing several repair cells derived from Remak cells in 4-week cut nerve of a PLP-Cre ERT2xRosa26stopYFP mouse. Scale bar, 30 μm. ***B***, Three examples of repair cells. Under each confocal image is an artificially colored (green) trace image of the same cell. Asterisks indicate the position of the nucleus. The cells are very narrow and elongated and have an undulating structure with occasional straighter stretches (e.g., type 2; middle). Top, Unbranched cell (type 1). Middle, Cell with two branches lying very close to each other on one side of the nucleus (type 2; enlarged part indicated by interrupted lines; arrow indicates the branch point). The two branches overlap toward the end of the cell. Bottom, Cell with long and irregularly curved branches on both sides of the nucleus (type 4; enlarged part indicated by interrupted lines; arrows indicate branch points). Scale bar, 30 μm. ***C***, Schematic drawings show a classification of repair cells depending on the presence of processes and their disposition. The box chart shows the frequency of each repair cell type in the total population of Remak-derived repair cells (as a percentage of total cells) at different times after injury. ***D***, Total percentage of branched cells among Remak cells and repair cells at different times after injury. Note that branching does not change significantly with time.

Extensive examination of nerves from tamoxifen-treated PLP-CreERT2xRosa26stopEYFP mice failed to reveal GFP-labeled cells other than the Schwann cells described above. To study the descendants of these cells in injured nerves, the sciatic nerve of such mice was cut and reinnervation prevented. After 7 d, 4 weeks, 10 weeks, and 6 months, the tibial branch of the sciatic nerve distal to the injury was examined after GFP/YFP immunolabeling (Fig. 1*B*,*D*). At the 4-week time point, these distal stumps contained scattered, very elongated, slender cells (Fig. 5*A*,*B*). Although the nerves were initially fixed *in situ* with the limb in extended position, the cells typically showed an irregularly curved or wavy appearance.

Unexpectedly, ∼50% of the cells were branched (Fig. 5*B*,*C*). The branches were often long and were always oriented parallel to the long axis of the cell. The branched cells could be divided into four types based on branching pattern, the most common pattern being the formation of two processes on one side of the cell (Fig. 5*C*). The average Remak-derived repair cell in 4-week cut nerves was 760.9 μm long, ranging from 249.0 to 1365.0 μm (Fig. 2*A*,*C*, Table 1). This means that, in addition to branching, Remak cells elongate on average 3-fold as they transform to repair cells. These repair cells are therefore ∼7-fold longer than immature Schwann cells.

In addition to the 4-week point described above, repair cells were also examined at other times after injury to test whether length of denervation affected length of repair cells (Fig. 2*C*, Table 1). We found that repair cells had not yet reached their full length 7 d after injury. More significantly, repair cells slowly shortened at long time points after injury, a period during which repair cells gradually lose their capacity to support axonal regeneration (Fig. 2*C*, Table 1; Sulaiman and Gordon, 2009). The number of cells that were branched remained, however, at ∼50% from 7 d to 6 months after injury (Fig. 5*D*, Table 2).

**Table 2.** Morphology

	Branching/type	%	*n*
PLP-CreERT2			
Repair Schwann 7 d after cut	Nonbranched/I	61.22	735
	Two branches/II	32.38	
	Three branches/III	1.90	
	Branches on both sides/IV	4.49	
Repair Schwann 4 wk after cut	Nonbranched/I	48.85	780
	Two branches/II	10.00	
	Three branches/III	4.36	
	Branches on both sides/IV	6.79	
Repair Schwann 10 wk after cut	Nonbranched/I	50.70	1004
	Two branches/II	37.55	
	Three branches/III	4.78	
	Branches on both sides/IV	6.97	
Repair Schwann 6 mo after cut	Nonbranched/I	51.03	243
	Two branches/II	40.74	
	Three branches/III	2.88	
	Branches on both sides/IV	5.35	
P0Cx-CreERT2			
Repair Schwann 4 wk after cut	Nonbranched/I	66.67	156
	Two branches/II	21.15	
	Three branches/III	1.28	
	Branches on both sides/IV	10.90	

### Repair Schwann cells derived from myelin cells

**Figure 6.**
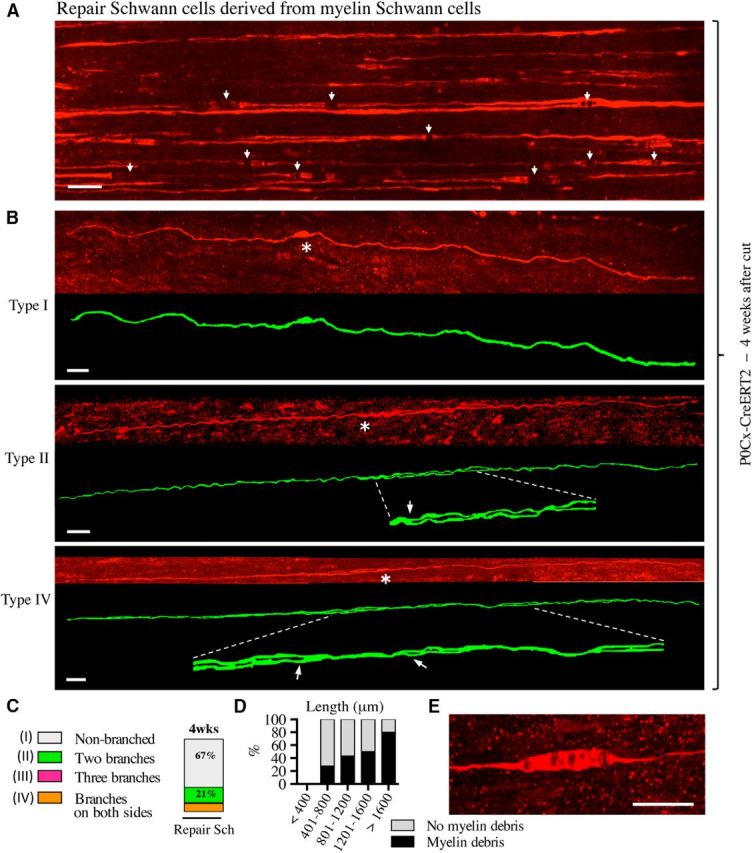
Myelin-derived repair cells. ***A***, Overview showing parts of a number of repair cells derived from myelin cells in 4-week cut nerve of a P0Cx-Cre ERT2xRosa26stopEYFP mouse. Some cells show swellings, which represent myelin debris and exhibit partial disruption of the fluorescence signal (examples indicated with arrows; see also ***D***, ***E***). Scale bar, 30 μm. ***B***, Three examples of repair cells derived from myelin cells. Under each confocal image is an artificially colored (green) trace image of the same cell. Asterisks indicate the position of the nucleus. The cells are extremely long and show a general appearance that is similar to that of Remak-derived cells. Top, Unbranched cell (type 1; see Fig. 5C). Middle, Cell with two branches on one side of the nucleus (type 2; enlarged part indicated by interrupted lines; arrow indicates the branch-point). Bottom, Cell forming branches on both sides of the nucleus (type 4; enlarged part indicated by interrupted lines; arrows indicate branch points). In both of the branched cells, the branches appear to be separate along a part of the cells only. This is caused by the two closely apposed processes overlapping at this angle of representation. Scale bar, 30 μm. ***C***, Frequency of each repair cell type in 4-week cut nerves. ***D***, Percentage of cells with myelin debris in different length groups of repair cells in 4-week cut nerves. Percentage of myelin-cell-derived repair cells that still contain undigested myelin increases with increased cell length. ***E***, Cose view of a swelling caused by undigested intracellular myelin debris. Scale bar, 20 μm.

**Figure 7.**
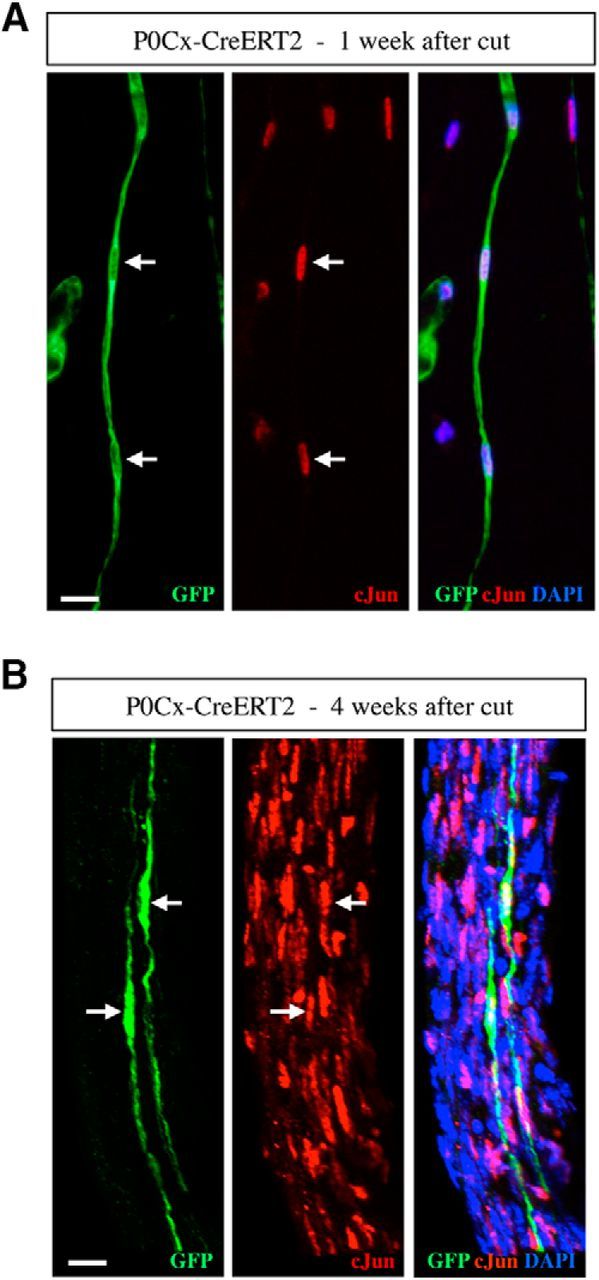
GFP-labeled repair cells express high levels of c-Jun. ***A***, ***B***, Teased nerve preparations made from 1- and 4-week cut nerves of tamoxifen-treated P0Cx-Cre ERT2xRosa26stopEYFP mice. The nerves are double immunolabeled with GFP and c-Jun antibodies. ***A***, Column (Bungner's band) of GFP-positive repair cells (left) 1 week after cut. The nuclei (arrows) are c-Jun-positive (middle). ***B***, Two GFP-positive repair cells (left). The nuclei (arrows) are c-Jun-positive (middle).

We did not detect GFP labeling in cells other than those clearly identifiable as myelin Schwann cells in uninjured tibial nerves of tamoxifen-treated P0Cx-CreERT2xRosa26stopEYFP mice. To determine the fate of these myelin cells after denervation, we examined the distal stump of the tibial nerve of such mice 4 weeks after transection without reinnervation (Fig. 1*B*,*D*). Extremely elongated cells were found dispersed throughout the distal stumps (Fig. 6). c-Jun is detectable at low, basal levels in some Schwann cells in uninjured nerves (Hantke et al., 2014; Klein et al., 2014), but is expressed at much higher levels in denervated Schwann cells, where it serves as a global amplifier of the repair phenotype (Arthur-Farraj et al., 2012; Jessen and Mirsky, 2016). We found that the elongated GFP-labeled cells in 4-week cut nerves showed strong nuclear c-Jun immunolabeling (Fig. 7).

**Figure 8.**
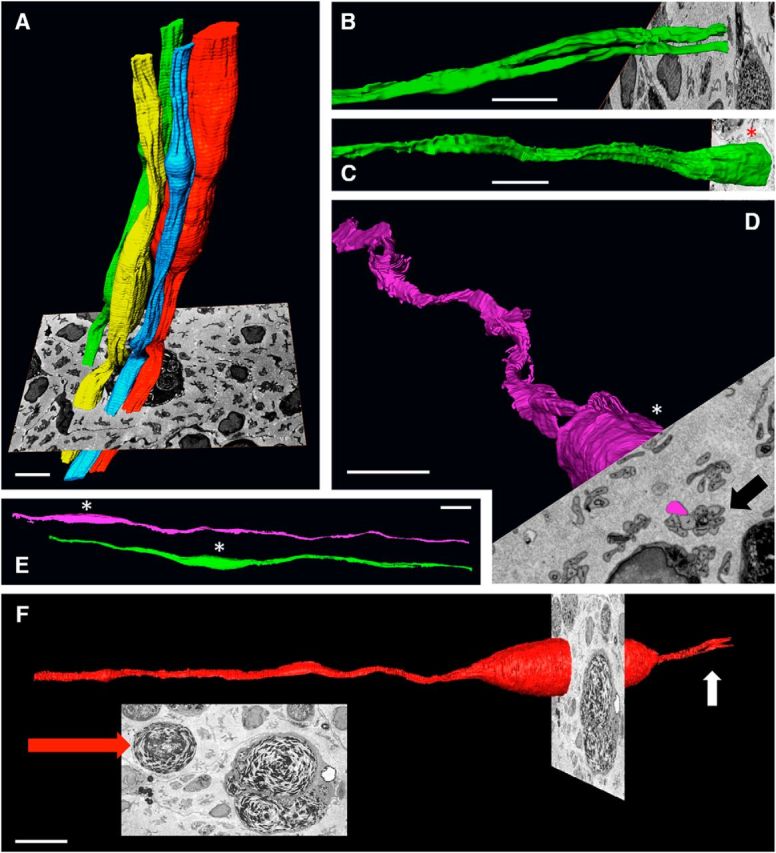
Block-face-scanning electron microscopy. ***A***, Tracing of whole Bungner's bands showing parts of four irregular cellular columns running longitudinally along the tibial nerve. Shown also is a 2D image of the block face from which they were generated. This shows transverse sections of several Bungner's bands, some of which cut through Schwann cell nuclei (arrow). Part of a myelin-containing macrophage can be seen behind the colored Bungner's bands. Scale bar, 10 μm. ***B***, Tracing of a single repair cell showing the point at which the cell branches, each branch lying in a separate Bungner's band. Scale bar, 10 μm. ***C***, ***D***, Part of two cells illustrating the spiraling, corkscrew-like structure typical of repair cells. In ***C***, the Bungner's band to which the cell belongs is seen in the 2D image of the block face (arrow). Scale bar, 10 μm. ***E***, Parts of two repair cells illustrating their undulating appearance. These cells belong to two neighboring Bungner's bands (view of the block face not shown). Scale bar, 10 μm. ***F***, Part of a repair cells containing a myelin lump. The scanning block face is shown face-on in the inset. The large red arrow points to the undigested myelin in the Schwann cell. A large macrophage also containing myelin is seen to the right of the Schwann cell. Small white arrow points to the point where the repair cell is starting to branch. Scale bar, 10 μm.

Although even longer, the general appearance of these cells was, in most respects, similar to that of Remak-derived repair cells and these cells could be classified in a similar way (Fig. 5*C*, Table 2). An exception to this was the presence of swellings along some cells in which the fluorescent signal was partly interrupted (Fig. 6*A*,*E*). They were identified as myelin debris (Fig. 8*F*). The percentage of cells containing myelin debris in 4-week cut nerves increased with cell length from ∼25% in the shortest ones to ∼80% among the longest cells (Fig. 6*D*). Schwann cells with myelin debris were more common at shorter times after injury (data not shown). These observations support the view that, as expected, the length of a repair cell reflects the length of the myelin cell from which it derived because clearing myelin likely takes a longer time in the case of a large myelin cell than a short one.

One-third of the repair cells were branched and the branching pattern was comparable to that seen among Remak-derived cells (Fig. 6*B*,*C*). These repair cells were approximately twice as long as the myelin cells from which they derived. Their average length was 1117.0 μm, with the longest cells measuring ∼2 mm (Fig. 2*A*,*D*, Table 1). Myelin-cell-derived repair cells therefore are ∼10-fold longer than immature Schwann cells in developing nerves (Fig. 2*A*,*B*,*D*).

### Examination of repair cells by block-face-scanning electron microscopy

To obtain a closer view of repair cells and Bungner bands, we used block-face-scanning electron microscopy to examine 4-week cut tibial nerves of WT mice. In these preparations, repair cells derived from Remak or myelin Schwann cells could not be distinguished. This is not of major concern because the observations described in previous sections did not reveal significant differences between these cell populations apart from their length and the occasional presence of myelin in the process of degradation in myelin-cell-derived repair cells.

The 3D visualization of Bungner's bands showed irregularly undulating parallel columns, reflecting the irregular appearance of their constituent cells (Fig. 8*A*). Tracing of individual cells showed that the branched cells frequently seen by light microscopy were also seen in these preparations and different branches of the same cell were sometimes seen to separate in such a way that they would be identified as belonging to two different Bungner's bands by conventional transmission electron microscopy (Fig. 8*B*). Typically, the elongated bodies of repair cells were wavy and often appeared to twist to form a shallow spiral (Fig. 8*C*,*D*). It can be speculated that this structure enables overlapping cells to braid or interweave closely to form a resilient cellular column. Striking localized expansions of the repair cell body were seen in some cells, corresponding to the structures seen by light microscopy (Fig. 6*E*). These were confirmed to be caused by a large lump of as-yet-undigested myelin debris showing characteristic whorls of parallel membranes (Fig. 8*E*), although smaller inclusions of myelin debris were also seen (data not shown).

### Conversion of repair cells to myelin cells after regeneration

**Figure 9.**
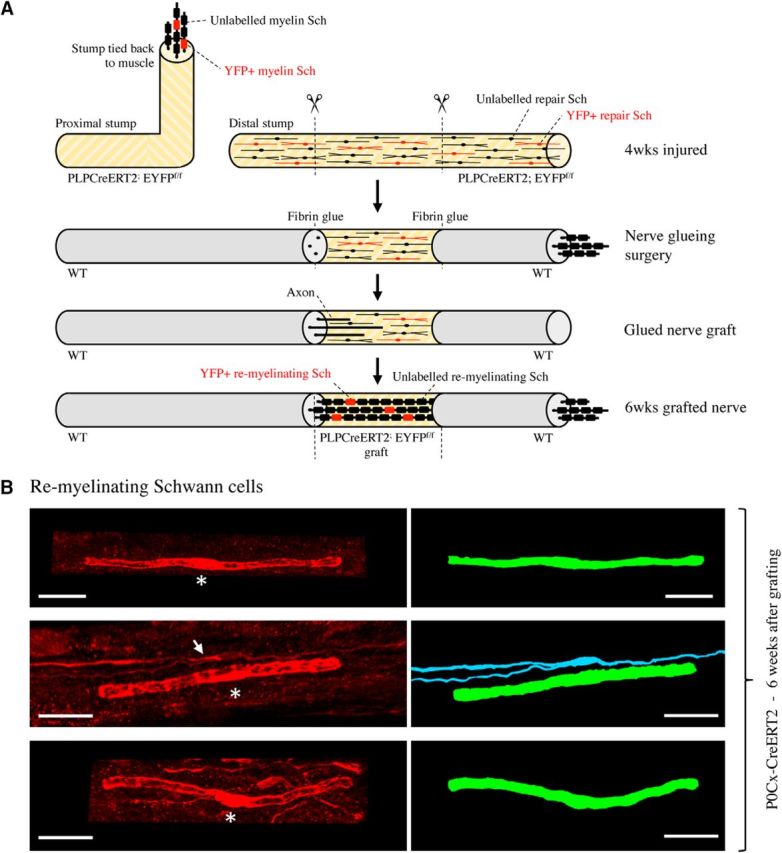
Remyelinating Schwann cells derive from repair cells. ***A***, Outline of nerve grafting experiments. ***B***, Three examples of the typically short remyelinating cells. These cells have formed myelin around axons in a 6-week nerve graft. Asterisks indicate position of the nucleus. Right, Artificially colored trace image of the same cells. The myelin cell in the middle panels lies close to a branched repair cell (arrow; blue), which has not converted to a myelin cell. Scale bar, 30 μm.

Direct evidence from lineage tracing has not been available for one of the basic tenets of Schwann cell biology: that denervated Schwann cells in injured nerves (repair cells) will, after regeneration, convert back to myelin Schwann cells, forming myelin sheaths around regenerated axons. We therefore traced the fate of the repair cells after they were allowed to reassociate with regenerating axons. For this, the sciatic nerve of tamoxifen-treated P0Cx-CreERT2xRosa26stopEYFP mice, in which only myelin cells are labeled, was cut and reinnervation prevented as described above. Four weeks later, the denervated tibial branch of the nerve containing GFP-labeled repair cells was transplanted to a WT mouse, where it was attached to a freshly cut tibial nerve and inserted as a bridge attached to both the proximal and distal end of the host nerve by fibrin glue (Fig. 9*A*). After allowing the tibial nerve of the host to regenerate through the transplanted stump for 6 weeks, the stump was removed, processed for GFP/YFP immunohistochemistry, and examined in the confocal microscope. This showed numerous GFP-labeled myelin Schwann cells exhibiting the characteristic Cajal bands and blunt ends at heminodes (Fig. 9*B*). Further, these cells showed the typical short length of myelin cells in regenerating nerves, measuring on average 141.1 μm, which is approximately one-quarter the length of myelin cells in uninjured nerves (Fig. 2*A*,*D*, Table 1). This is consistent with published observations (Hildebrand et al., 1985), whereas the length or Remak cells in regenerating nerves has not been studied. Because the only GFP-labeled cells we could see in 4-week cut tibial nerves were the very elongated, often branched repair cells described earlier, we conclude that these cells convert to the short myelin Schwann cells identified in regenerated tibial nerves in the present experiments. A surprising implication of this is that repair cells shorten radically, amounting to ∼7-fold on average, as they convert from the repair phenotype to the myelin phenotype (Fig. 2*A*).

## Discussion

It has been known for a long time that distal to nerve injury, myelin, and Remak cells reorganize to form columns of elongated cells, bands of Bungner, which are indispensable for nerve regeneration. However, our knowledge of the structure of these regeneration tracks and the cells that form them has not advanced for decades.

In the present work, first, we show that Schwann cells start a process of striking elongation and often elaborate long branches when they lose axonal contact after nerve injury. This results in cells that are approximately 2-fold and 3-fold longer than the myelin and Remak cells, respectively, from which they originate. This Schwann cell elongation is consistent with the extension of processes by terminal Schwann cells in response to denervation (Reynolds and Woolf, 1992; Son and Thompson, 1995). Second, we show that immature Schwann cells in developing nerves are unbranched and significantly shorter than myelin and Remak cells, measuring ∼1/5 and 1/2 of their length, respectively. This means that repair cells are on average ∼9-fold longer than the immature Schwann cells seen in E18 nerves. Therefore, the structure of denervated Schwann cells is characteristic of the repair cell state and this cell-type-specific morphology differentiates repair cells from other cells in the Schwann cell lineage. Third, we show that the transition of repair cells to myelinating cells is accompanied by a remarkable shortening, by ∼7-fold, to generate the typically short myelin Schwann cells well known to populate regenerated nerves. Cell shortening does of course not take place as immature cells transit to myelin cells during development. Fourth, although myelin and Remak cells have strikingly different structure, they give rise to repair cells with similar morphology. The general appearance of myelin- and Remak-cell-derived repair cells and their branching pattern are comparable and the two populations show extensive overlap in size, although myelin-cell-derived repair cells are on average longer and sometimes contain myelin debris. Fifth, the present experiments provide the first direct evidence using genetic lineage tracing for two basic assumptions in Schwann cell biology: that myelin and Remak cells generate the elongated cells that build Bungner bands in injured nerves and that such cells can transform to myelin cells after regeneration.

### Implications of repair cell structure

The structure of repair cells revealed here is tailored to generate uninterrupted and robust cellular tracks for regenerating axons because their length and parallel processes maximize the degree of cell–cell overlap within each Bungner band. The typical spiraling of the cell body is also likely to help generate a tightly interwoven cell column. A 2- to 3-fold increase in cell length in response to injury provides the Schwann cell injury response with a further safety factor because it ensures the formation of continuous regeneration tracks made from overlapping Schwann cells even without the need for Schwann cell proliferation. This may help to explain the unexpected observation that, after crush injury, the nerve extent of axon growth appears to regenerate normally in mice in which Schwann cell proliferation is inhibited (Kim et al., 2000; Atanasoski et al., 2001; Yang et al., 2008). Incidentally, this finding also suggests that the reprogramming of Remak and myelin cells to functional repair cells may not depend on reentry to the cell cycle. The signals that induce Schwann cell proliferation after injury remain poorly understood (Murinson et al., 2005; Atanasoski et al., 2008; Fricker et al., 2013).

### Injury region versus the distal stump

The repair cells that we have studied in the present work are the cells that occupy nerves distal to injury (the distal stump) along which axons need to grow, often for long periods of time, to reconnect with their targets. These cells may differ from the Schwann cells that occupy the injury region itself after complete nerve transection. This region contains at least two populations of Schwann cells (Jessen et al., 2015b; Cattin and Lloyd, 2016). The first group are the cells that accompany axons in the regeneration units that extend from the injured proximal nerve toward the distal nerve stump. Most of these cells have not lost contact with axons and for that reason are likely to differ from repair cells. The second group are other Schwann cells that migrate out from the distal stump toward the proximal stump, thereby, unlike repair cells, losing contact with the basal lamina. These two Schwann cell populations, together with fibroblasts, macrophages, and other cells, form a tissue bridge that connects the proximal and distal stumps. The length of this bridge is minimized by surgical repair, which brings the proximal and distal stumps into close proximity.

### Repair cell maintenance

Maintenance of the repair-supportive features and survival of repair Schwann cells are central issues for the success of nerve regeneration. Because axons grow slowly, Schwann cells in the more distal parts of nerves in larger animals including humans remain denervated for long periods. During this time, the denervated cells gradually lose their capacity for supporting axonal growth and their number declines. This deterioration of nerves distal to injury has been modeled and documented in rodents and is considered a major reason for regeneration failure in humans (Sulaiman and Gordon, 2009, 2013; Eggers et al., 2010; Scheib and Höke, 2013). The mechanisms that control the phenotypic instability of repair cells are therefore of great practical importance as well as biological interest. It is therefore surprising that examination of the relevant signaling mechanisms is just starting. Recently, the transcription factor STAT3 has been implicated in the long-term maintenance of repair cells (Benito et al., 2017). Injury triggers STAT3 activation in Schwann cells, which is sustained during chronic denervation. In mice with selective inactivation of STAT3 in Schwann cells, long-term denervated nerves contain fewer Schwann cells, express reduced levels of repair cell markers and trophic factors, and have Bungner's bands with distorted structure. In contrast, Schwann cell development is normal. This suggests a specific role for STAT3 in Schwann cells, which is to support the survival of chronically denervated Schwann cells and maintain their long-term molecular and morphological differentiation.

### Shortening of repair cells: a comparison with developmental myelination

The myelin cells that form in regenerated nerves are short, measuring only ∼1/4 the length of myelin cells in uninjured nerves (Hildebrand et al., 1985). This was confirmed in the present study, which found that GFP-labeled myelin cells averaged 576 μm in uninjured nerves, whereas the length of GFP-labeled myelin cells in regenerated nerves was 150 μm. This size is strikingly similar to the length of early myelin cells starting to elaborate myelin sheaths during development (Court et al., 2004). Developing Schwann cells subsequently elongate to match the increase in nerve length as the animal grows, perhaps because of passive stretching (Court et al., 2004). Because this does not apply to adult regenerated nerves, the length of their myelin Schwann cells should reflect cell length near the onset of myelination. Intriguingly, this suggests that, in both uninjured nerves of neonates and regenerated nerves of adults, Schwann cells start myelination at a comparable length of ∼100–150 μm. The observation that repair cells undertake a radical shortening to only ∼13% of their original length to achieve this length as they myelinate regenerated axons suggests that this length is optimal for the onset of myelination.

### Reprogramming versus de-differentiation

The fact that the structure of denervated Schwann cells is unambiguously different from both Schwann cells in developing nerves and cells in the lineage gives additional support to the notion that denervated Schwann cells represent a distinct Schwann cell differentiation state. Historically, these cells have been characterized either as activated or de-differentiated Schwann cells (Jessen and Mirsky, 2016). Because the Schwann cell injury response in fact combines the activation of repair-supportive phenotypes with de-differentiation (loss of the myelin phenotype), we have argued that it represents cellular reprogramming, resulting in the generation of a distinct cell, the repair cell, which is specialized to support regeneration (Jessen et al., 2015a; Jessen and Mirsky, 2016). In addition to the cell-type-specific morphology shown here, this cell performs a number of functions specifically related to injury and repair and are under distinct transcriptional controls, exemplified by c-Jun, STAT3, and H3K27 trimethylation. All of these have a role in repair cells, but not in Schwann cell development (Arthur-Farraj et al., 2012; Jessen and Mirsky, 2016; Ma and Svaren, 2016; Benito et al., 2017).
